# Obituary

**Published:** 1910-09-15

**Authors:** 


### OBITUARY.

Dr. Frank S. Buckley died at his residence in Oak Park, Monday, January 17, after a five days' severe illness developing a complication of troubles on Sunday afternoon, with which Dr. Willard and Dr. Roberts and two nurses fought with all their medical skill, the patient himself struggling with wonderful tenacity to maintain his hold on life.

Dr. Buckley was born September 1, 1857, in Milwaukee. His boyhood was spent in Manistee, Mich., where he attended the grade and high schools. He spent two years in the naval academy at Annapolis. He was a graduate of Oberlin college and of the dental department of the University of Michigan. In 1890 he went to Berlin, Germany, becoming associated with the late Dr. Miller and practicing his profession there three years. He then went to Beirut, Syria, remaining there six months. In 1894 he returned to this country and resided here until the year 1900, leaving then for Constantinople, Turkey, where he practiced dentistry for six years. He then came back to Chicago in 1906 and practiced here until his death.

Dr. Buckley is survived by his widow and one son, Harold N. Buckley He was a brother of Edwar Buckley, and of Mrs. Elizabeth J. Wing, both of Manistee, Mich.

The funeral services were held at the First Congregational church Thursday at 3 o'clock. The remains were taken to Manistee, Mich., for interment.

Died at Columbus, Ohio, June 19, 1910, of Aortic Regurgitation, W. H. Todd, D.D.S., in his fifty-seventh year.

Dr. Todd was born in Columbus, Ohio, May 10, 1854, and received his preliminary education in the public schools of that city. At an early age he became associated with his father, Dr. Hiram Todd, who was the first dentist in Columbus, coming to this city from New York City more than seventy years ago. He was graduated in 1882 from the Ohio College of Dental Surgery and continued in the practice established by his father at the old home residence in South High Street, where he enjoyed the confidence and esteem of a large and discriminating clientele, for he was a most pains-taking and skillful dentist, being always in the front rank of the progressive ones. His relation to the dental profession was most cordial. He was kind, courteous and ethical to the highest degree, a prince among gentlemen.

He was a member of the Ohio State Dental Society, always taking a lively interest in its procedings and an active part in its government, having served on practically every important committee. Was chairman of the Arrangements Committee for seven years, where he did valiant service for the society. Was president of the society in 1895, and was a member of the Board of Directors from 1891 to the time of his death; was also a member of the Northern Ohio Dental Society, Columbus Dental Society, National Dental Association and an associate member of the New York Institute of Stomatology. He was also largely instrumental in founding the Ohio Dental Library Association, of which he was made president; a society that succeeded in collecting one of the largest dental libraries in the world and having for its permanent home an alcove in the public library at Columbus, Ohio, where it becomes a fitting and practical monument to his memory.

In 1884 he married Miss Flora Barcus, of Columbus, who with two sons, William and Henry, survive him.—W. I. JONES.—In *Summary*.

